# New Experimental Equipment Recreating Geo-Reservoir Conditions in Large, Fractured, Porous Samples to Investigate Coupled Thermal, Hydraulic and Polyaxial Stress Processes

**DOI:** 10.1038/s41598-018-32753-z

**Published:** 2018-09-28

**Authors:** C. I. McDermott, A. Fraser-Harris, M. Sauter, G. D. Couples, K. Edlmann, O. Kolditz, A. Lightbody, J. Somerville, W. Wang

**Affiliations:** 1 University of Edinburgh, School of Geosciences, The King’s Buildings, James Hutton Road, Edinburgh, EH9 3FE Scotland; 2 Heriot-Watt University, Institute of Petroleum Engineering, Edinburgh, EH14 4AS Scotland; 3 University of Göttingen, Centre of Geosciences, Goldschmidtstr. 3, 37077 Göttingen, Germany; 4 Helmholtz Centre for Environmental Research – UFZ, Department of Environmental Informatics, Permoserstraße, 15/04318 Leipzig, Germany; 5 Applied Chair Environmental System Analysis, Technische Universität Dresden, Dresden, Germany

## Abstract

Use of the subsurface for energy resources (enhanced geothermal systems, conventional and unconventional hydrocarbons), or for storage of waste (CO_2_, radioactive), requires the prediction of how fluids and the fractured porous rock mass interact. The GREAT cell (Geo-Reservoir Experimental Analogue Technology) is designed to recreate subsurface conditions in the laboratory to a depth of 3.5 km on 200 mm diameter rock samples containing fracture networks, thereby enabling these predictions to be validated. The cell represents an important new development in experimental technology, uniquely creating a truly polyaxial rotatable stress field, facilitating fluid flow through samples, and employing state of the art fibre optic strain sensing, capable of thousands of detailed measurements per hour. The cell’s mechanical and hydraulic operation is demonstrated by applying multiple continuous orientations of principal stress to a homogeneous benchmark sample, and to a fractured sample with a dipole borehole fluid fracture flow experiment, with backpressure. Sample strain for multiple stress orientations is compared to numerical simulations validating the operation of the cell. Fracture permeability as a function of the direction and magnitude of the stress field is presented. Such experiments were not possible to date using current state of the art geotechnical equipment.

## Introduction

Multi-physics process-based understanding of the subsurface (0–4 km depth) is increasingly enabling society to address many significant challenges through existing and newly developing technology. Examples include low and high temperature geothermal energy extraction techniques^1,2^, recovery of conventional and unconventional hydrocarbons^3^, the storage isolation of substances such as CO_2_ and toxic radioactive waste^4–11^, energy storage^12,13^ and the subsurface injection of liquid wastes^14,15^. Sustainable management of the subsurface requires the ability to understand, predict and monitor the physical response of the geo-reservoirs and the surrounding rock mass to changes in fluid pressure, stress, temperature, fluid composition and biological activity. These physical responses are often described as combinations of thermal (T), mechanical (M), hydraulic (H), chemical (C), and micro-biological processes (B). All of these processes are interdependent to some degree, with feedbacks and degrees of coupling among themselves that depend on the particular situation and technology under consideration.

Experimental approaches provide an opportunity to gain better understanding of process interactions and provide quantitative values that underpin the development and calibration of behavioural laws. A primary motivation for the experimental apparatus development reported here is to progress the capability for experimental investigation of coupled THM processes in geo-reservoirs under *in-situ* condition controls that are beyond the current state of the art. The design goals for this development emphasise large samples to allow more complexity of the fracture(s) within the sample, the capability of achieving rotatable, true-triaxial stress states, the ability to heat the sample in the apparatus and the ability to investigate fluid flow through the sample under controlled conditions. To date this has not been possible within a single experiment. This paper describes the new apparatus and initial operational outcomes that demonstrate its capabilities.

## Previous Work and Context

Understanding the behavior of rocks under representative stress states has long been a goal of rock mechanics research. The first so-called triaxial testing equipment, the “von Kármán cell”^16^ enabled the investigation of brittle and ductile rock failure. In the von Kármán cell, a uniform axi-symmetric radial loading is exerted by means of a pressurized fluid in the annulus between the walls of the pressure cell and the sample. The design served as a blue print for many variations to follow, with the “Hoek-Franklin cell”^17^ becoming widely adopted in the field of rock mechanics, along with similar cell designs^18,19^. The International Society of Rock Mechanics (IRSM) describe the use of such cells as a standard for rock failure testing^20^.

However these conventional triaxial cells, are strictly only able to create conditions that are representative of two specific triaxial stress states^21–26^.

1 $${\sigma }_{1} > {\sigma }_{2}={\sigma }_{3}$$

2 $${\sigma }_{1}={\sigma }_{2} > {\sigma }_{3}$$

Actual measurements indicate that the three principal stresses are rarely equal in the natural setting^27,28^. Analytical and numerical calculations show that the general condition is one of “true triaxial” conditions, in which the principal stresses are unequal:

3 $${\sigma }_{1} > {\sigma }_{2} > {\sigma }_{3}$$

The intermediate principal stress has an important influence on the criterion that defines rock failure and both normal and shear stress across fracture plains, which in turn significantly influences the fluid flow properties of the rock mass^19,29,30^. This emphasises the need to represent true triaxial 3D stress conditions, and led to the development of true triaxial testing (TTT) equipment^31,32^ (and references therein). In a review of the history of TTT apparatuses^21^, three main categories of testing equipment are identified, (i) rigid platen type, (ii) flexible medium type, and finally (iii) mixed type. Almost all TTT testing relies on cubic samples, and most have a sample size with an edge length of under 10 cm. All cubic samples are limited to one orientation of the principal stress axes with respect to discontinuities and/or strength anisotropy, though the actual magnitude of the stresses may be varied e.g.^33–38^.

A number of new TTT cells have been developed for specific purposes, for example the visual observation of deformation of prismatic samples through a sapphire window^39^, hydraulic fracturing of differently sized cubic specimens^40,41^, and rapid unloading to simulate rock burst conditions^42–46^. A TTT cell has been developed capable of containing larger samples for testing enhanced geothermal systems (EGS) with a side wall dimension of 300 mm and enabling the whole cycle of an EGS development, from drilling multiple boreholes during heating and mechanical loading through to production and post-production^47^. Larger samples (500 mm × 500 mm × 500 mm) to capture representative elementary volume (REV) scale results have also been investigated^48^. However, samples larger than 300 mm side length require specialist preparation and handling equipment due to their weight, and multiple sample testing in the laboratory is challenging.

Fluid flow in the subsurface occurs both in the matrix pore system and fractures. Understanding flow under different stress conditions is important: current TTT apparatus facilitates measurements of effective permeability, pore pressures up to 35 MPa, elevated temperatures (of up to 200 °C), as well as imaging of the sample through both P and S wave velocities, acoustic emission monitoring, and electrical resistivity^36^. Other designs allow further fluid sealing^49^ and increased loading capacity^50^. Commercial true tri-axial testing apparatus products, such as TerraTek hydraulic fracturing cells, DCI Test Systems’ polyaxial stress frame, or Wille-Geotechnik’s advanced true triaxial test system, are also available and commonly used to investigate rock strength and hydraulic fracturing^51,52^.

To the knowledge of the authors, there is only one example of a cell capable of applying a rotatable stress field to cylindrical samples with a diameter of 38 mm and length of 76 mm, the SMART cell^24,53,54^. The use of cylindrical specimens has a significant advantage over the cubic samples since it reduces the concentration of stresses at the sample edges overcoming the problem of the blank loading corners^21,33^. However, the issue of end effects due platen friction leading to a stress shadow where the vertical loading plates contact the top and bottom surfaces of the sample is still important, and taken into account in the evaluation of the stress field developed in the sample through modelling and strain measurement.

The GREAT cell (Geo-Reservoir Experimental Analogue Technology) presented here represents a mixed type “polyaxial cell” capable of creating principal stresses from multiple directions, and even irregular distributions of stress without the need to re-position the sample. The GREAT cell represents a significant advancement in testing capability with respect to sample size, stress control and monitoring technology. The technological development embodied in the cell progresses the radial pressure concept illustrated by the SMART cell, with a new radial hydraulic pressure system that can accommodate more sample displacement and higher loading than was possible in the SMART cell. It involves a complete redesign of the radial loading concept and mechanics, ensuring minimal interference between pressure exerting elements. The GREAT cell is designed to recreate *in situ* conditions found at depths of 3 to 4 km in geo-energy applications in terms of triaxial polyaxial stresses to 100 MPa, temperatures up to 100 °C and fluid pressure up to 40 MPa with flow. The sample size of 0.2 m diameter facilitates the investigation of fracture networks, with specially-positioned fluid ports selecting specific fluid flow channels/features within the samples. Rotation of the stress field during experiments enables investigation of the behavior of fractures and fracture networks under changing loading conditions. The strain response and, in future experiments, the temperature response, of the samples are monitored through the use of state of the art fibre optic cable providing thousands of detailed all around measurements. Multiple pressure sensors provide detailed real time stress and fluid pressure measurements of the loading applied.

We demonstrate the operational capability of the cell for two different synthetic samples, a homogeneous sample and a sample hydraulically fractured between two artificial boreholes. The first set of experiments is the simplest possible, with a homogenous material demonstrating mechanical deformation. The second is relevant to hydraulic fracturing for enhanced geothermal systems, where a doublet of wells is being engineered, but also to fracturing for shale gas where such a short-cut is undesirable, and where each subsequent hydraulic stimulation will change the stress orientation on the fractures in the preceding fracture stage.

In the second sample, flow is induced between the boreholes in a stress field that is rotated, allowing the flow changes as a function of stress to be determined. To our knowledge, this has not been achieved before. The aim of investigating artificial samples is to demonstrate that the GREAT cell could (a) exert a controllable, rotatable poly-axial stress field, (b) facilitate detailed surface strain monitoring of the surface deformation of the sample and (c) contain fluid flow with considerable backpressure through samples in a rotating stress field.

The homogeneity of the undeformed artificial sample allows the deformation of the sample and subsequent strain response to be benchmarked against standard mathematical models for elastic behaviour. Experimental testing is undertaken over a period of a few hours with repeated loading and unloading performed in different orientations thereby reducing the possibility of longer term visco-plastic deformation of the synthetic material used (polyester). In the future, the repeatability of measurements will also enable the plastic and elastic responses of the rock under cyclical loading and different stress states to be investigated. Understanding the mechanical impact of more frequent cyclic loading/unloading operations is particularly important for energy storage in subsurface systems. Strain on the surface of the sample is determined using optical fibre technology, providing high-resolution spatial data, and providing a possible strain value (axial or circumferential) at a spacing of 2.5 mm along the length of the fibre. The low elastic modulus of the sample material (~4 GPa, compared to natural rocks that are typically 20 GPa+) constrains the stress magnitudes that can be achieved with the artificial material because radial deformation of more than 2–3 mm on a sample with a radius of 100 mm would compromise the integrity of the optical fibre. The current testing was undertaken with a rotating triaxial stress field (σ_*x*_ > σ_*y*_) from 2 MPa to 10 MPa, (equivalent to the rock stress expected at 100 to 500 m depth) and with fluid in the fracture at pressures up to ~4 MPa (~400 m depth). This amount of deformation can be considered equivalent to a test of ~40 MPa true triaxial stress on a rock sample, which can be mapped to rock stress exerted at a depth of ~1.5 km. Numerical modelling of the deformation and comparison to the circumferential strain measurements indicate that the cell is able to create a rotatable true triaxial stress field and that deformation of the sample surface can be accurately recorded.

A significant difference in circumferential-surface deformation of the unfractured and hydraulically fractured samples is observed, even where the fracture has not propagated to the surface of the sample. Rotating the stress field allowed investigation of the impact of the fracture orientation on the surface deformation, relative to the external principal stress directions. Within a numerical model of this experiment, a generic fracture of similar geometry to that within the sample gives results that match the observed deformation, illustrating that deformation processes occurring deep in the sample can be detected remotely at the sample surface using the fibre optic monitoring equipment.

Fluid flow was induced through the fracture under different stress orientations by flowing through two boreholes connected to the fracture. Permeability of the fracture is found to be a function of the normal stress across the fracture plane^55^ (and references therein), a result further confirming the operation of the cell.

The GREAT cell was then used to raise the temperature of the sample by some 20–30 °C in an unconfined state, and allowed to cool down again after which the fluid flow experiment was repeated with a fracture fluid pressure of 3.2 MPa, simulating raised pore pressures. Heating led to some plastic deformation of the fracture and a reduction in permeability.

## Design of the GREAT cell

**Figure 1:**
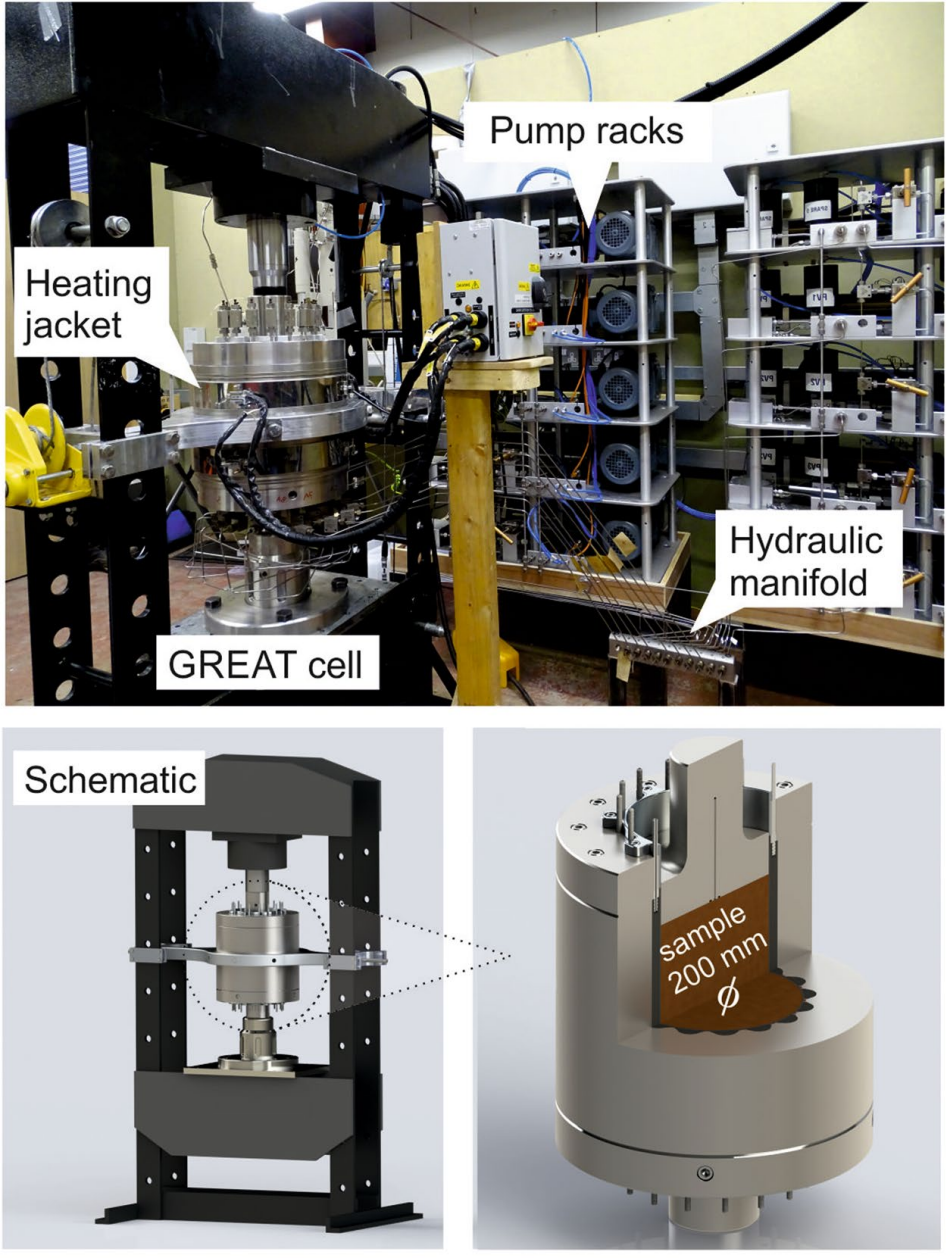
GREAT cell experimental apparatus.

The GREAT cell (Fig. 1) is designed to accommodate large, bench-scale cylindrical samples (approximately 200 mm diameter × 200 mm length) and to subject them to conditions of temperature, pressure, and fluid pressure representative of subsurface conditions, including true triaxial stress conditions. As such, the design criteria require a full working range of 100 MPa radial and axial loading, temperatures of up to 100 °C, and pore pressures up to 40 MPa. In addition, the poly-axial design criterion necessitated the individual control of the radial loading mechanism.

**Figure 2:**
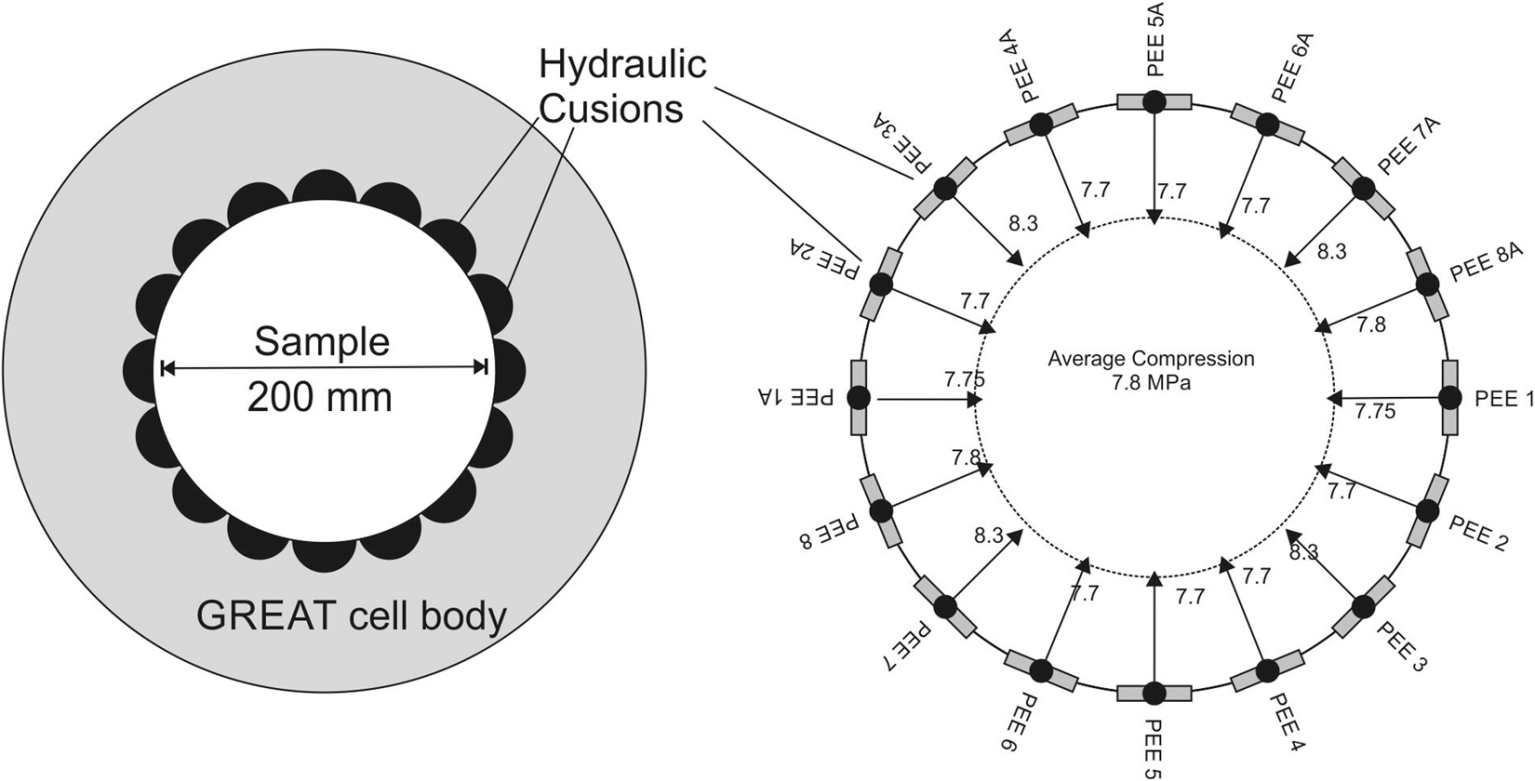
Concept of hydraulic cushions exerting a radially controlled stress field on a sample with a diameter of 200 mm, PEE labelling refer only to experimental notation, numbers with arrows refer to fluid pressure in PEE during a particular experiment given as an illustration.

The radial pressures are applied to the sample by eight opposing pairs of fluid filled flouro-elastomer (Viton) tubes, forming hydraulic cushions, termed Pressure Exerting Elements (PEEs). The PEEs have a large-radius face that matches the sample diameter, and a short-radius back that fits into recesses in the cell body (Fig. 2). The PEE pairs are connected to automatically controlled pressure generator pumps. Each PEE pair is connected hydraulically and pressurised by the same pump ensuring the same pressure is exerted symmetrically on opposite sides of the sample.

Individual PEEs are prevented from influencing the pressure in the neighboring PEEs by a Dynamic Sealing Strip (DSS). Currently a 2mm-thick Viton sheath is placed between the sample and the PEEs/DSSs to protect the fibre optic cable attached to the sample and to ensure a complete hydraulic seal around the sample during fluid flow experiments.

Fluid flow through a sample is achieved with a separate hydraulic system controlled by LabVIEW and supported by separate pumps. Fluid enters and leaves the cell through the top platen via two fluid ports to allow fracture flow-through tests – one central port and a second at a radius of 50 mm. The platen design allows the use of a distribution plate to ensure fluid access to parts or the whole of the sample’s end surface. It is also possible to record the temperature of the fluid entering and leaving the sample through two in-line thermocouples on the inlet and outlet flow lines.

Sample temperature is controlled using electro-resistance heating bands located circumferentially around the main cell body, spaced evenly in the axial direction. An insulating jacket encompasses the whole cell including the plumbing and the hydraulic loading ram. Thermal breaks are used between the cell body support and the loading frame.

Strain data on the sample deformation is recorded using a fibre optic strain gage along with the ODiSI-B software produced by LUNA Inc. This fibre enables an extremely high resolution of fibre-parallel strain to be determined around the cylindrical surface of the sample: e.g., a 1 m length cable corresponds to an equivalent of 461 possible strain gage points. A groove <1 mm deep was cut into the sample surface into which the optical fibre for measuring strain was attached.

## Experimental Program

We present the results of three different experimental programs, listed in Table 1. All further data is available from the corresponding author on request.

**Table 1:** Unique aspects of experimental tests.

Test name	Unique aspect of test	Description of sample
Mechanical Test (M1)	Mechanics only Rotating triaxial stress field	Homogeneous artificial sample Opaque amorphous thermoplastic polymer
Hydromechanical Test (HM1)	Hydraulics and Mechanics Fluid flow through fracture in rotating triaxial stress field stress field	Artificial hydraulically fractured sample Transparent polyester resin
Hydromechancial Test (HM2)	As HM1, with significant fracture fluid pressure (4 MPa)	As HM1 after being further thermally fractured in cell Transparent polyester resin

**Figure 3:**
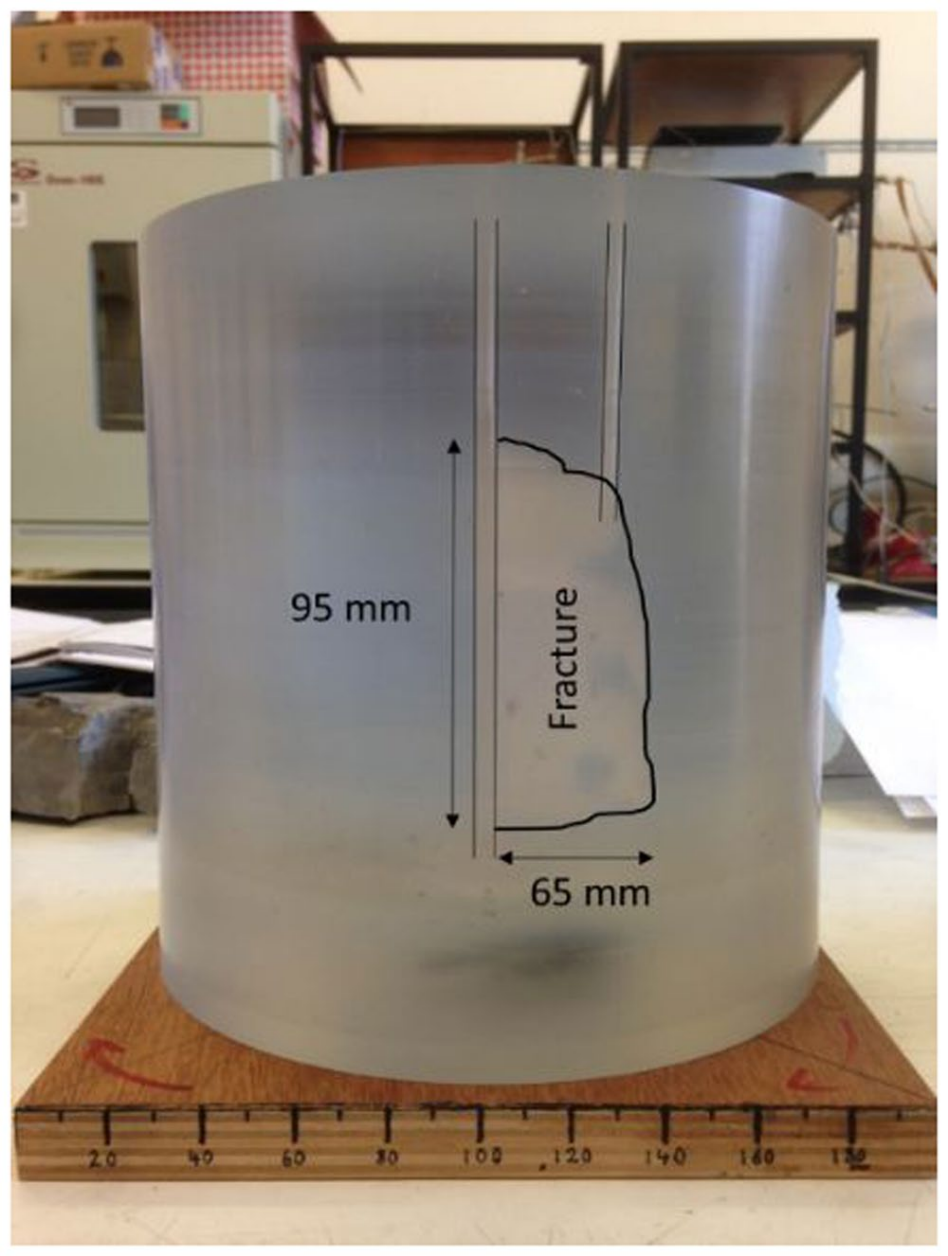
The prepared sample showing the location of the fracture (edge is highlighted on the photo) and boreholes in HM1 & HM2 experiments.

The sample for the mechanical tests M1 is made from an opaque amorphous thermoplastic polymer, whilst the sample for the fracture flow tests (HM1 & HM2) was made from a transparent polyester resin. A uniaxial compression hydraulic fracturing rig was used to generate a hydraulic fracture within sample HM1 through a central borehole. A second borehole was then drilled into the sample to create a dipole fluid flow scenario within a confined fracture of dimensions 95 mm high × 65 mm width (Fig. 3). Once the HM1 tests were complete, the sample was heated in the GREAT cell, and afterwards found to have thermally fractured and thus the fracture extended to the cylindrical margin of the sample. This sample then formed the basis for the test sequence HM2.

The samples were loaded first with a vertical stress of 8.2 MPa in the M1 test and 10 MPa in the HM tests, representing σ_1_. Then for all tests, initially an equal radial compression is applied through the PEEs, and then the PEEs are adjusted to create a triaxial stress field with σ_1_ > σ_2_ > σ_3_, of the order of 8 MPa for σ_2_ to 2 MPa for σ_3_. A detailed listing of the PEE pressures for the experiments presented here in each test stage for M1, HM1 and HM2 is given the Supplementary Information. The fracture flow tests of HM1 and HM2 were conducted under different flow conditions, HM1 had a flow rate of 30 ml/min with the downstream pressure being ambient atmospheric conditions, HM2 was conducted at a flow rate of 25 ml/min with a fluid downstream back pressure of 3.45 MPa. In each case, flow rate and fluid pressure were recorded at a frequency of 1 Hz, and sample-surface strain was recorded at 25 Hz.

## Benchmarking the Experimental Results by Numerical Modelling

To assess whether the GREAT cell is creating the internal stress field expected, the operation of the cell is simulated using the open source coupled THMC processes simulator OpenGeoSys (www.opengeosys.org). Modelling a deforming 3D body with multiple traction terms, boundary conditions, multiple layers and time dependent application of stress is a non-trivial exercise. The theory and several benchmarks regarding the use of OGS may be found in^56^, and general FE theory found in^30^. Here, the numerical calculation of the elastic deformation and surface strain is compared to the measured deformation of the samples during the experiments.

**Figure 4:**
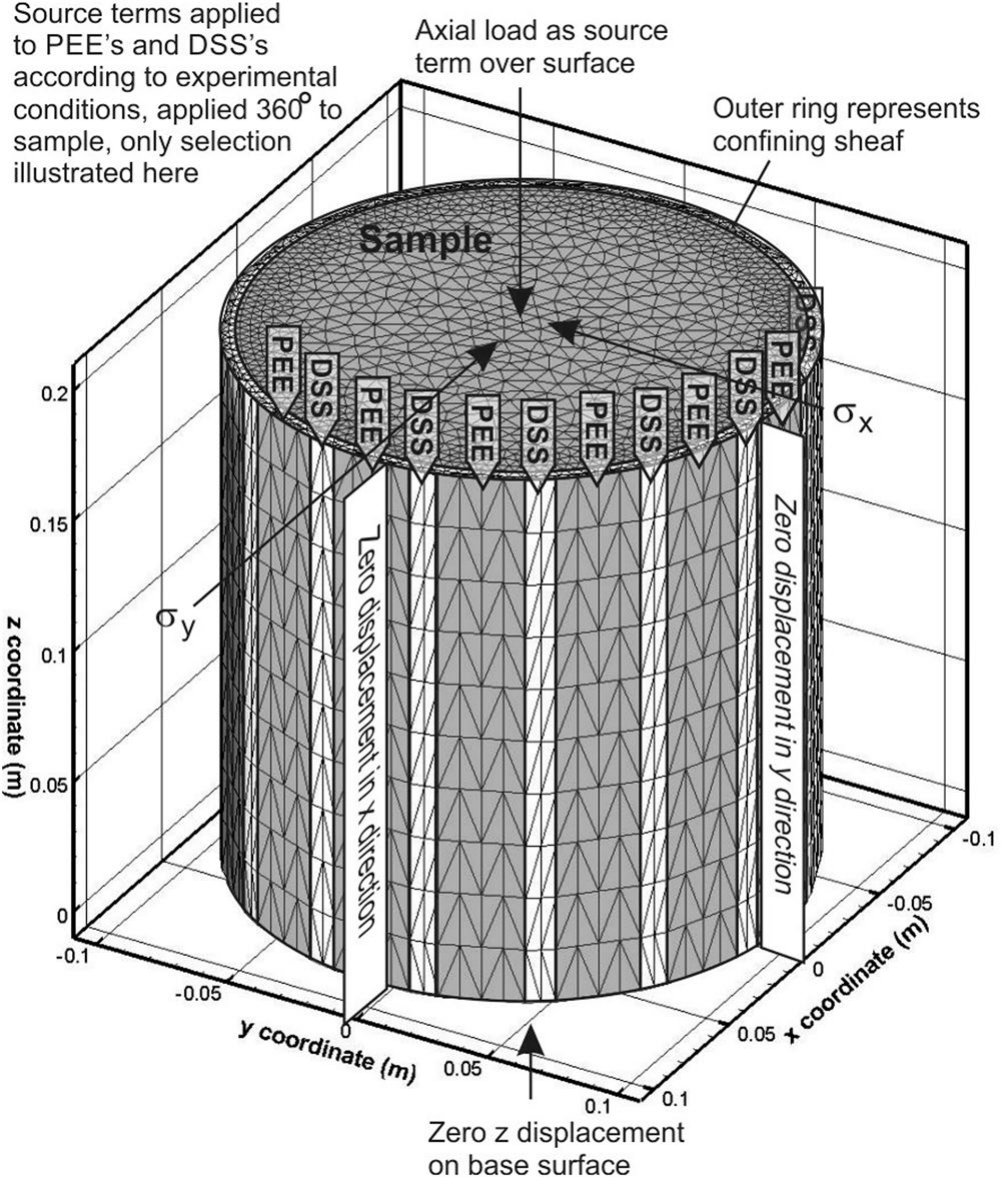
Conceptual numerical model, illustrating mesh, selection of boundary conditions and application of source terms to represent experimental conditions.

A cylindrical structured mesh representing the sample was created using Gmsh software^57^. The mesh geometry and density were designed to ensure that nodes correspond to key sample geometrical features. For multi-stage experiments involving axial loading, followed by true triaxial radial loading and then rotation of those loads, stage-dependent traction loads are applied. We define zero circumferential-displacement boundary conditions along the vertical lines that define the sample circumference intersection with the x- and y-axes, and a zero displacement in the z-direction across the entirety of the sample base (Fig. 4). This simulates the sample assuming no end effects though end plate friction. To include the possibility of endplate friction, the worst possible case is simulated by defining zero displacement in the x- and y- directions across the entirety of the sample top and base.

To simulate the experiments HM1 and HM2 it was necessary to represent the fracture in the numerical mesh. Elements belonging to the fracture volume have a sub-millimetre thickness and are assigned an elastic modulus significantly less (>10 x reduction) than the surrounding intact material, thereby representing a softened region in the elastic continuum.

Three models are presented here. Each model simulates both the axi-symmetric stress field σ_1_ > σ_2_ = σ_3_ and the true triaxial stress field σ_1_ > σ_2_ > σ_3_. The models were realised by varying stage-dependent traction terms, boundary conditions, material properties or meshes as reflecting the experimental conditions. Model 1 simulates the experimental test M1, Model 2 simulates tests HM1&2, but without taking into account the presence of the fracture, and Model 3 simulates HM1 & HM2 with a discrete fracture included in the numerical mesh. The elastic material properties used to simulate all tests are given in Table 2. The actual samples used for M1 and HM1&2 were made from different materials, thus the elastic parameters are different, although all are representative of literature values for these materials.

**Table 2:** Parameters of elastic models used to simulate GREAT cell results.

Sample	Model 1	Model 2	Model 3
	(M1)	(HM1&2)	(HM1&2)
Parameter			
Sample Youngs Modulus (GPa)	3.85	4.3	4.3
Sample Poisson Ratio	0.4	0.4	0.4
Fracture Youngs Modulus (GPa)			0.3
Fracture Poisson Ratio			0.4
Containing Sheaf Young Modulus (GPa)	0.1	0.1	0.1
Containing Sheaf Poisson Ratio	0.4	0.4	0.4

## Results

**Figure 5:**
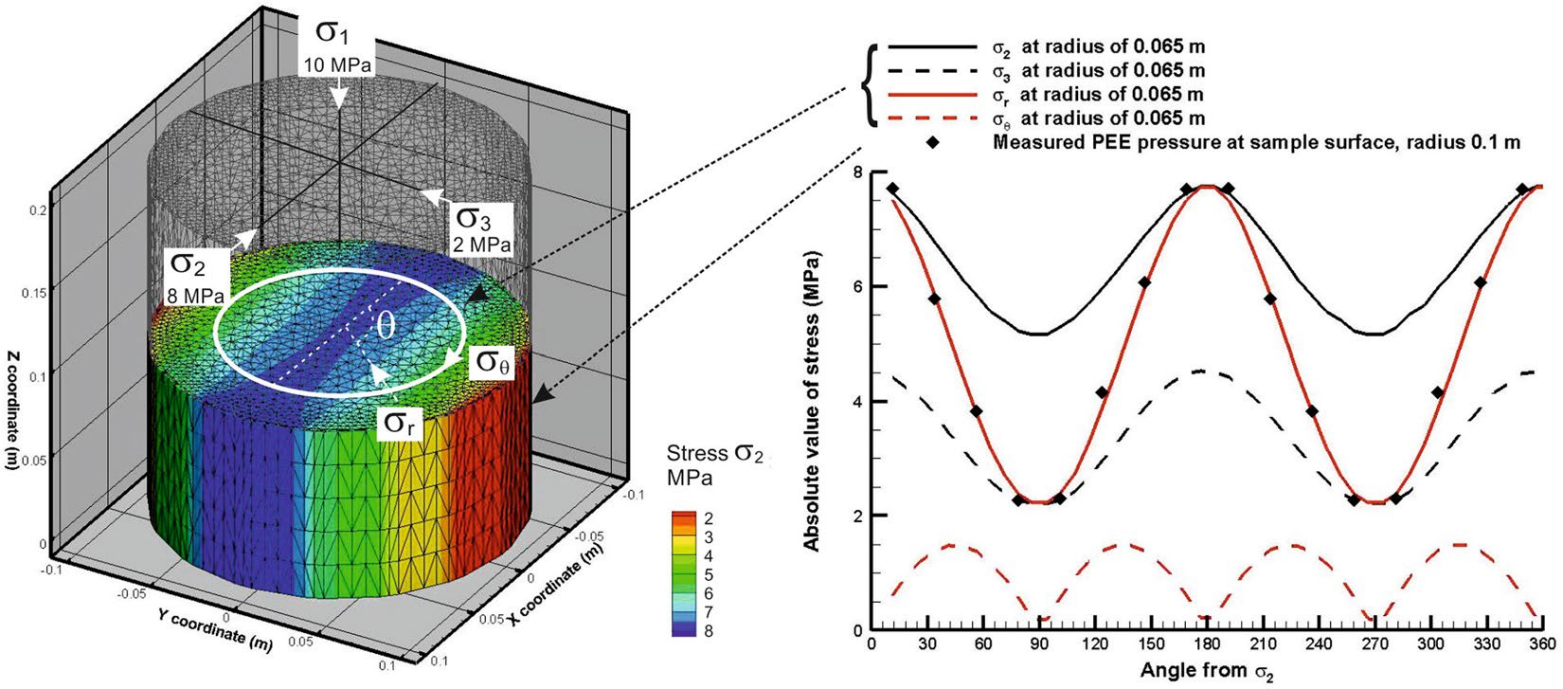
Numerical simulation of the stress field in M1 test, σ1 > σ2 > σ3, graph gives stress at locus of points shown by white circle at radius 0.065 m, PEE pressures measured at sample surface.

The results of the simulation (for the loading case: σ_1_ = σ_axial_ = 10 MPa, σ_2_ = 8 MPa, and σ_3_ = 2 MPa) for an ideal sample with no top surface or bottom surface boundary friction effects (“friction free sample”) are illustrated in Fig. 5. The simulation shows that the loading scheme creates an almost-correct and almost-homogeneous true-triaxial state in the sample volume. The small discrepancies between the ideal target state and the one that can be achieved are associated with the inability to apply shear tractions on the curved exterior surface. In the experiment, the sample circumference is everywhere a principal plane (as it is in all similar experimental designs), whereas a true-triaxial state would resolve onto planes with those orientations as a combination of normal and shear tractions. For the worst case end effect due to friction on the endplate a small reduction in the intensity of the stress field at the radius of 0.065 m is calculated of the order of <0.5%. The maximum difference noted in the center of the sample was of the order of 15% for the minimum principal stress, and 4% for the maximum principal stress. Although the average surface strains for the two models are within 4%, the period of the strains are shifted by 90 degrees, providing a useful way to determine the relative influence of any end effects. It is clear from these results that controlling the pressure in the PEEs facilitates a controlled triaxial stress field with user defined orientation within the sample.

**Figure 6:**
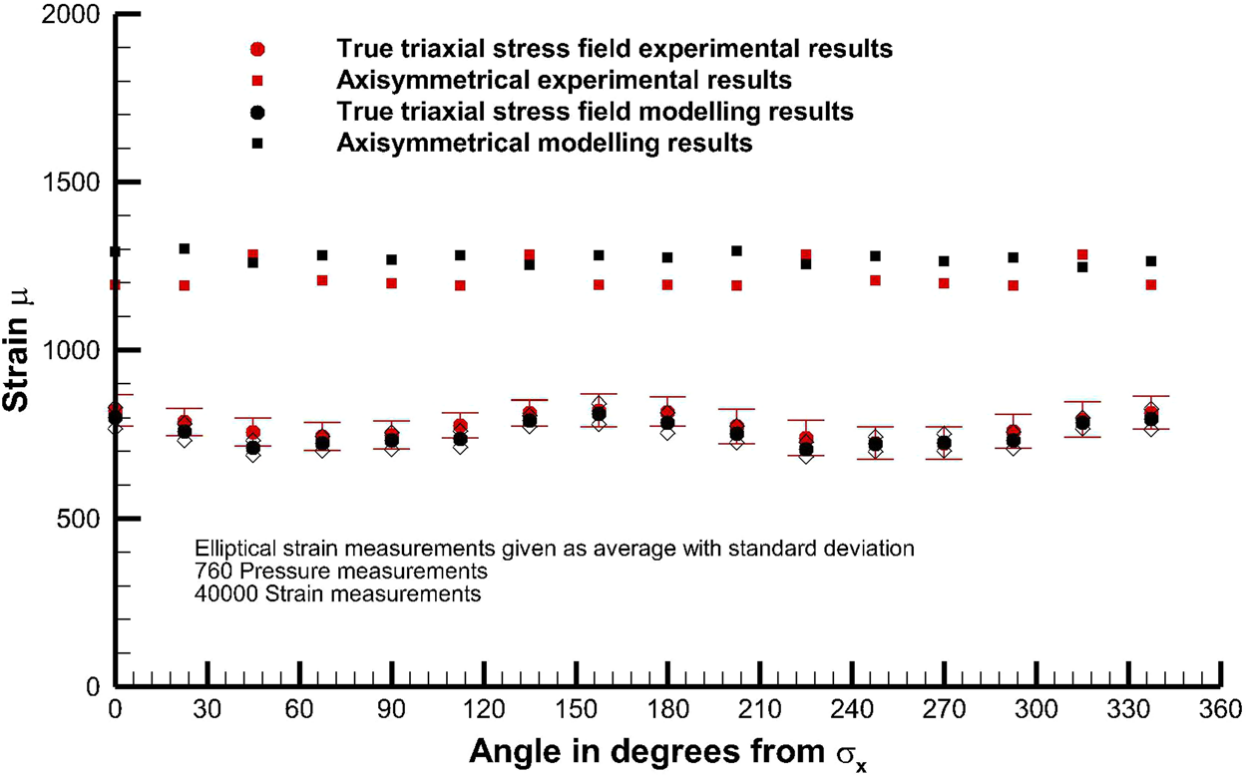
Comparison between experimentally measured strain and model simulations for Test M1, bars give standard deviation.

The measured and modelled surface strains for experiment M1 show a good consistency (Fig. 6). Here, 80 pressure measurements and 4000 strain measurements for the axi-symmetric radial loading case (σ_1_ > σ_2_ = σ_3_) are presented, as well as 760 pressure measurements of PEE pressure and 40000 measurements of strain for the true triaxial loading case (σ_1_ > σ_2_ > σ_3_). The model used superimposes with equal weighting a friction free sample and the worst end effect possible. The results demonstrate that the actual experiment performs very closely to the design. The true triaxial stress field measurements are shown as superimposed results derived from multiple physical orientations of the stress field on the actual sample. That is, a certain number of measurements were made with the stress field located in a certain orientation, the stress field was then rotated, and the strain then re-measured, repeated for 8 different orientations of the stress field. The results are presented relative to the orientation of the experimental principal stress axes.

Strain measurement locations on the sample were selected at 16 locations at the centre of the PEEs, halfway up the sample, with a radial interval of 22.5°. The mesh density of the numerical model enabled extraction of strain every 2.5°, i.e. with a much higher resolution than experimentally recorded, in addition linear interpolation between adjacent points gives a value of strain exactly corresponding to the location of the measurement. The surface strain is determined by the local change in length in the fibre optic cable. This is associated with a combination of the circumferential and radial strain components inside the sample itself.

**Figure 7:**
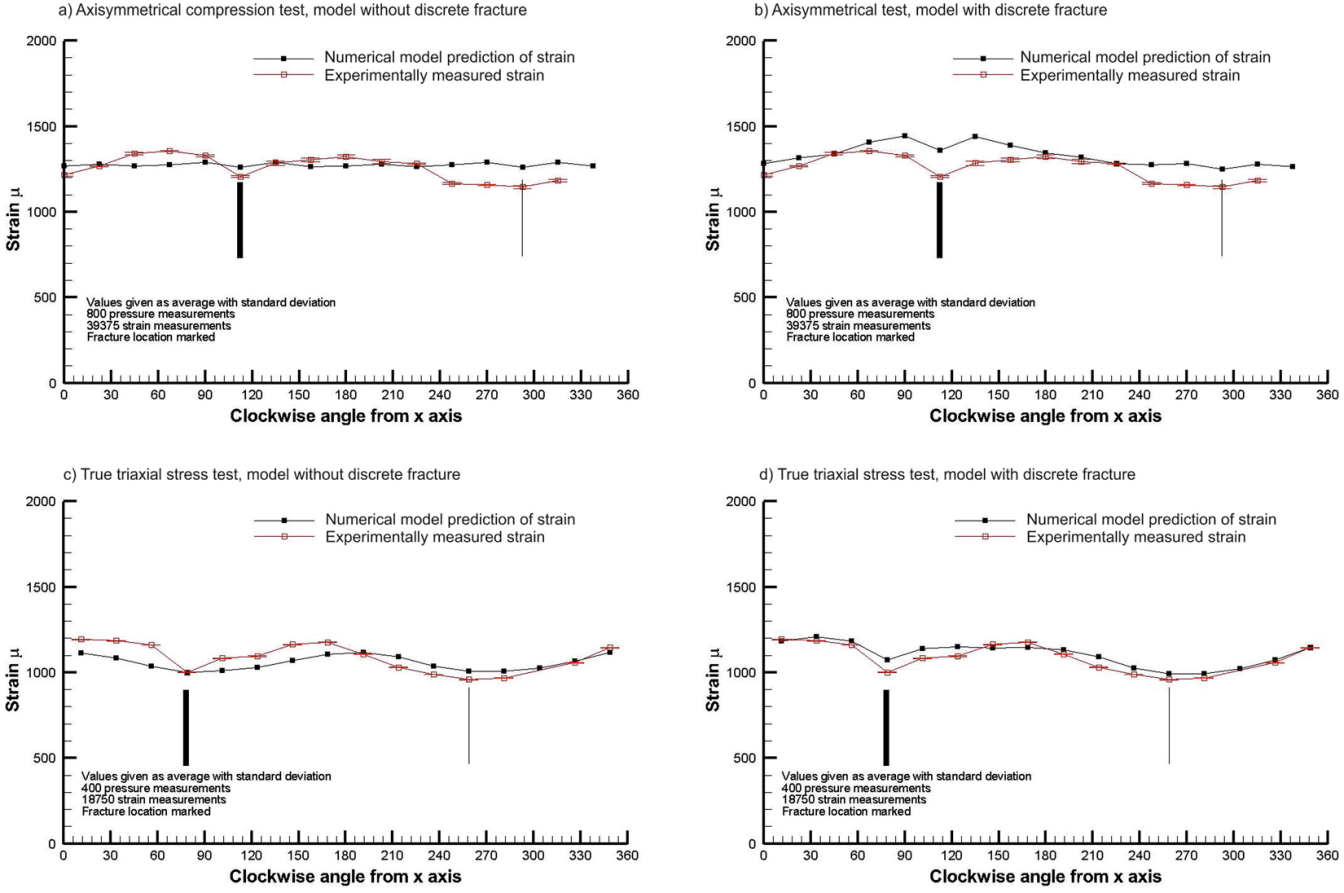
Comparison of the experimentally measured surface strains of a sample with a discrete fracture in it with model predictions with and without the inclusion of a discrete fracture in the mesh, for the axisymmetric radial compression test and a true triaxial stress field test.

Figure 7a illustrates the results of experiment HM1 assuming friction free conditions without explicitly including a fracture (Model 2), and leads to a moderate match of the surface deformation profile (within 250 µ) for the simpler axisymmetric (σ_1_ > σ_2_ = σ_3_) loading. This can be compared with the good match (within 100 µ and profile) depicted in the upper part of Fig. 6 for M1, for which both experiment M1 and Model 1 do not contain a fracture. Immediately obvious in Fig. 7a is that the location of the fracture (thick black line) in the sample is having a significant influence on the surface strain distribution. When a fracture is included in the numerical mesh (Model 3), illustrated in Fig. 7b, the match is significantly improved, clearly indicating that the fracture is the dominant factor determining the change in the surface strain when compared to the case where there is no fracture.

For the true triaxial loading case, Fig. 7c illustrates the comparison of the measured surface strain with the prediction of the numerical model without a discrete fracture in the mesh and Fig. 7d depicts the prediction with a discrete fracture in the mesh. The comparison of these results suffices to illustrate the GREAT cell is capable of detecting the surface strain expression of the fracture in a sample, and that numerical modelling is a viable means of constraining some important sensitivities to the fracture properties and orientation, which can be fitted to the observations obtained from the GREAT cell.

In both test sequences HM1 and HM2, the relationship between the orientation of the principal stress axes and the flow properties of the fracture could be investigated. The normal stress $({\sigma }_{n})$ across the fracture was calculated from the orientation and magnitude of the principal stresses assumed if true-triaxial conditions are achieved inside the sample, using the orientation of the fracture plane relative to these axes (Equation 4).

4 $${\sigma }_{n}={l}^{2}{\sigma }_{1}+{m}^{2}{\sigma }_{2}+{n}^{2}{\sigma }_{3}$$

where the orientation of the fracture to the principal stress axes is described by the directional cosines *l*, *m*, *n*.

The fluid pressure and flow rate through the fracture was recorded as a function of loading. The results for the fluid flow measurement in HM1 and HM2 are presented in the Supplementary Information. For the HM1 tests no downstream fluid pressure was applied, whereas for HM2 a downstream fluid pressure ~3.2 MPa was maintained. The permeability of the fracture was evaluated (Equation 4), and the effective aperture of the fracture determined. The fracture aperture *e* (m) is calculated using the cubic law approximation^58^ where *Q* (m^3^/s) is the volumetric flow across the sample through the boreholes, $\frac{{\rm{\Delta }}P}{{\rm{\Delta }}x}$ (Pa/m) is the pressure gradient between the boreholes, µ (Pa.s) is the dynamic viscosity, *w* is the fracture width (0.095 m, see Fig. 3) and Δ*x* is the fracture length (0.05 m see Fig. 3).

5 $$Q=w\frac{{e}^{3}}{12}\frac{1}{\mu }\frac{{\rm{\Delta }}P}{{\rm{\Delta }}x}$$

The intrinsic permeability k (m^2^) of the fracture is calculated from the fracture aperture *e* (m) as

6 $${\rm{k}}=\frac{{e}^{2}}{12}$$

**Figure 8:**
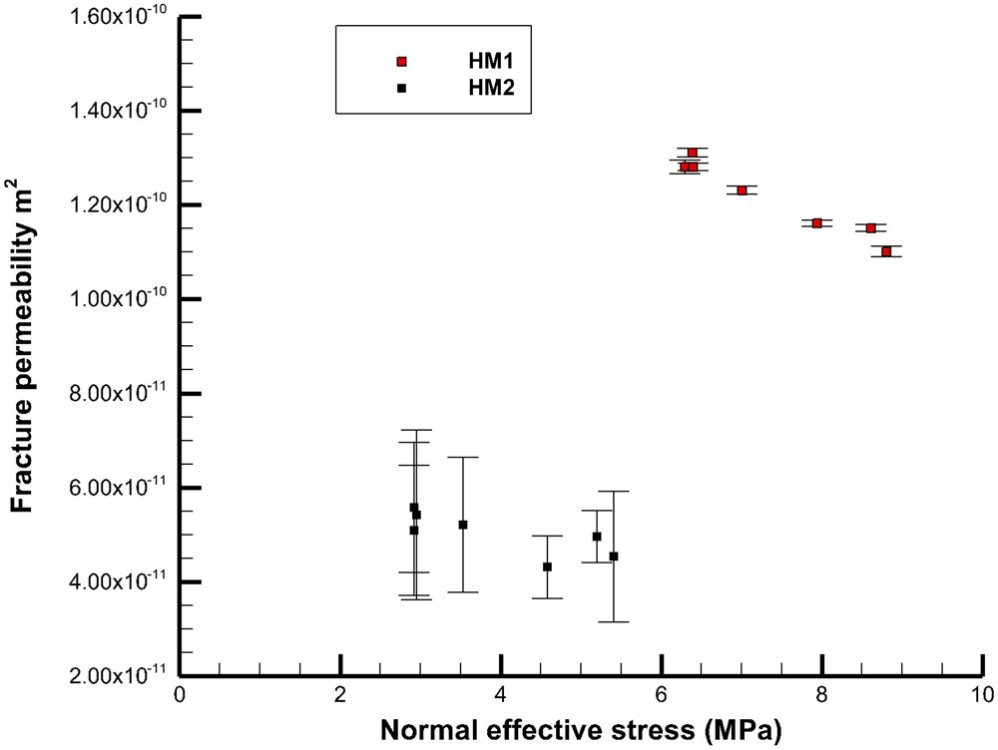
Plot of effective normal stress and change in permeability for fractures in HM1 & HM2 (HM1 376 measurements, HM2 460 measurements, error bars give standard deviation).

Fracture permeability is plotted against the calculated normal stress across the fracture plane, depending on the orientation of the stress field (Fig. 8). The results demonstrate a clear and consistent change in the permeability of the fractures with the change in the orientation of the stress field, and the change in normal stress on the fracture plane due to the rotation of the stress field. These results demonstrate that the GREAT cell can be used to investigate the flow effects of contained fractures inside a large sample. The increased error in the measurement of the HM2 experiments over the HM1 experiments is related to the extra pump control required to maintain a significant downstream pressure.

## Conclusions

The GREAT cell has the operational capacity to recreate representative reservoir conditions of a true poly-axial stress state/field in 200 mm diameter samples. This capability facilitates studies of fluid flow through fractures (and, in the future, investigation of coupled processes in fractured-porous media, fracture-+matrix samples interaction), and will provide opportunities to extend the range of parameters and conditions which are considered important in fracture-dominated flow. Optical fibre cable sensing of surface strain facilitated thousands of detailed strain measurements that provide good constraints for numerical models of the internal processes. There is an excellent correlation between imposed loading conditions, presence and character of the fracture in the sample, and material behavior. Dipole fluid flow through a fracture accessed by two artificial boreholes proves the fluid sealing capability of the cell. During fluid flow the triaxial stress field was rotated causing the normal stress across the fracture to change. There is a clear proportional relationship between the resolved normal stress and the effective flow in the fracture. The GREAT cell provides a step change in technology to experimentally investigate the coupled process behavior of natural reservoir material under *in situ* conditions of temperature, fluid flow, stress and chemistry. In particular, this technology advances the ability to investigate large diameter samples including fractures under *in situ* conditions of stress and temperature, to rotate a true triaxial stress field during a flow experiment, to provide fracture and matrix flow measurement with and without downstream pressure, and to provide thousands of detailed real time strain measurements on the surface deformation of the sample.

## Electronic supplementary material

Supplementary Information

## Data Availability

We present the results of three different experimental programs, listed in Table 1, within the manuscript and Supplementary Information. Original experimental data (0.25 GB), and model simulations (2.8 GB) is available from the corresponding author on request.
