# Supplementary material for: New Experimental Equipment Recreating Geo-Reservoir Conditions in Large, Fractured, Porous Samples to Investigate Coupled Thermal, Hydraulic and Polyaxial Stress Processes
Source: Sci Rep. 2018 Sep 28;8:14549. doi: 10.1038/s41598-018-32753-z (PMC6162306; doi:10.1038/s41598-018-32753-z)
Supplement: Supplementary file 1 — Supplementary Information [file 41598_2018_32753_MOESM1_ESM.pdf]

SUPPLEMENTARY NFORMATION For

New Experimental Equipment Recreating Geo-Reservoir Conditions in Large, Fractured, Porous Samples to Investigate Coupled Thermal, Hydraulic and Polyaxial Stress Processes.

***C. I. McDermott[1], A. Fraser-Harris[1], M. Sauter[3], G. D. Couples[2], K. Edlmann[1], O. Kolditz[4,5], A. Lightbody[1], J. Somerville[2], W. Wang[4]**

[1] University of Edinburgh, School of Geosciences, The King's Buildings, James Hutton Road, Edinburgh EH9 3FE.

[2] Heriot-Watt University, Institute of Petroleum Engineering, Edinburgh EH14 4AS

[3] University of Göttingen, Centre of Geosciences, Goldschmidtstr. 3, 37077 Göttingen

[4] Helmholtz Centre for Environmental Research – UFZ, Department of Environmental Informatics, Permoserstraße 15 / 04318 Leipzig / Germany.

[5] Applied Chair Environmental System Analysis, Technische Universität Dresden, Germany.

Corresponding author: Christopher McDermott (christopher.mcdermott@ed.ac.uk)

Supplementary Information

Symmetrical and triaxial stress fields for Test M1 on homogeneous sample, σ1 is the vertical axial load, σ2 and σ3 are in the horizontal plane.

	PEE 1	PEE 2	PEE 3	PEE 4	PEE 8A	PEE 7A	PEE 6A	PEE 5A
Angle PEE 1 to σ2 (°)	(MPa)	(MPa)	(MPa)	(MPa)	(MPa)	(MPa)	(MPa)	(MPa)
σ2=σ3	7.71	7.70	8.30	7.80	7.74	7.70	8.30	7.72
-11.25	7.73	5.70	4.39	2.40	2.30	4.00	6.40	7.70
11.25	7.70	7.73	5.70	3.70	2.00	2.30	4.28	5.70
33.75	6.20	7.73	7.70	5.72	3.70	2.10	2.29	3.90
56.25	4.26	6.18	7.70	7.70	5.72	3.70	2.29	2.30
78.75	2.27	4.30	6.30	7.70	7.72	5.71	3.70	2.30
-78.75	2.30	2.29	4.30	6.17	7.72	7.73	5.71	3.70
-56.25	3.70	2.30	2.38	4.20	6.10	7.73	7.72	5.70
-33.75	6.38	4.18	2.46	2.32	4.01	5.28	7.64	7.66
-11.25	7.76	5.70	3.70	2.30	2.30	4.20	6.27	7.71

Symmetrical and triaxial stress fields for Test 2 HM1 on a fractured sample, fluid flow through fracture without in situ fracture fluid pressure. PEE 7A is in the plane of the fracture, σ1 is the vertical axial load, σ2 and σ3 are in the horizontal plane.

	PEE 1	PEE 2	PEE 3	PEE 4	PEE 8A	PEE 7A	PEE 6A	PEE 5A
Angle PEE 1 to σ2 (°)	(MPa)	(MPa)	(MPa)	(MPa)	(MPa)	(MPa)	(MPa)	(MPa)
σ2=σ3	8.80	8.80	9.20	9.00	8.90	8.70	9.30	8.70
-56.25	7.10	6.30	6.30	6.90	7.90	8.70	8.80	7.80
33.75	7.70	8.70	8.70	7.70	7.30	6.30	6.30	6.60
56.25	7.20	8.20	8.70	8.70	7.80	6.80	6.30	6.20
78.75	6.20	7.20	8.30	8.70	8.70	7.70	6.90	6.30
-11.25	6.20	6.20	7.30	8.10	8.70	8.70	7.80	6.70
67.5	6.80	6.40	6.90	7.50	8.20	8.70	8.30	7.20

Fluid pressure measurements for different normal stresses across a fracture generated by rotating a triaxial stress field in Test 2 HM1

Normal Stress	Inlet fluid pressure	Outlet fluid pressure	Normal Eff. Stress	dP/dx	Flow Rate	Perm. *k*	Ap. *e*
MPa	MPa	MPa	MPa	MPa/m	ml/min	m^2	μm
8.81	1.0094	0	8.81	1.3123	30	1.10E-10	36.38
6.39	1.0102	0	6.39	1.3132	30	1.31E-10	39.61
8.61	1.0107	0	8.61	1.3140	30	1.15E-10	37.08
7.94	1.0115	0	7.94	1.3149	30	1.16E-10	37.39
7.01	1.0120	0	7.01	1.3157	30	1.23E-10	38.41
6.30	1.0125	0	6.30	1.3162	30	1.28E-10	39.26
6.40	1.0128	0	6.40	1.3166	30	1.28E-10	39.15

Symmetrical and triaxial stress measurements for Test 3 HM2 on a fractured sample, fluid flow through fracture with in situ fracture fluid pressure of 3.43 MPa, PEE7A is in the plane of the fracture, $\sigma 1$ is the vertical axial load, $\sigma 2$ and $\sigma 3$ are in the horizontal plane.

	PEE 1	PEE 2	PEE 3	PEE 4	PEE 8A	PEE 7A	PEE 6A	PEE 5A
Angle PEE 1 to $\sigma 2$ (°)	(MPa)	(MPa)	(MPa)	(MPa)	(MPa)	(MPa)	(MPa)	(MPa)
$\sigma 2 = \sigma 3$	8.72	8.73	9.13	8.73	8.72	8.72	9.24	8.71
-56.25	7.20	6.27	6.29	7.10	7.90	8.72	8.70	7.90
33.75	7.70	8.74	8.71	7.70	7.20	6.28	6.29	6.71
56.25	7.20	8.10	8.80	8.76	7.80	6.70	6.28	6.30
78.75	6.23	7.29	8.21	8.74	8.68	7.68	6.94	6.12
-11.25	6.20	6.30	7.24	8.20	8.78	8.72	7.80	6.80
-56.25	6.80	6.20	6.30	7.20	8.20	8.80	8.70	7.70

Fluid pressure measurements for different normal stresses across a fracture generated by rotating a triaxial stress field in Test 3 HM2, with an in situ fracture pressure of 3.43 MPa

Normal Stress	Inlet fluid pressure	Outlet fluid pressure	Normal Eff. Stress	dP/dx	Flow Rate	Perm. *k*	Ap. *e*
MPa	MPa	MPa	MPa	MPa/m	ml/min	m^2	μm
8.84	3.62	3.43	5.41	3.90	23.50	4.53E-11	23.32
6.38	3.58	3.43	2.95	3.06	24.05	5.42E-11	25.49
8.63	3.61	3.43	5.20	3.62	24.95	4.96E-11	24.39
8.01	3.61	3.43	4.58	3.59	20.06	4.31E-11	22.73
6.96	3.60	3.43	3.53	3.35	24.93	5.21E-11	25.01
6.35	3.58	3.43	2.92	3.01	21.57	5.09E-11	24.71
6.35	3.58	3.43	2.92	3.04	24.98	5.58E-11	25.87
